# Supplementary material for: Human Parainfluenza Virus (HPIV) Detection in Hospitalized Children with Acute Respiratory Tract Infection in the Western Cape, South Africa during 2014–2022 Reveals a Shift in Dominance of HPIV 3 and 4 Infections
Source: Diagnostics (Basel). 2023 Aug 2;13(15):2576. doi: 10.3390/diagnostics13152576 (PMC10417174; doi:10.3390/diagnostics13152576)
Supplement: Supplementary file 1 [file diagnostics-13-02576-s001.zip › diagnostics-2507439-supplementary.pdf]

Supplementary data for HPIV paper 2023

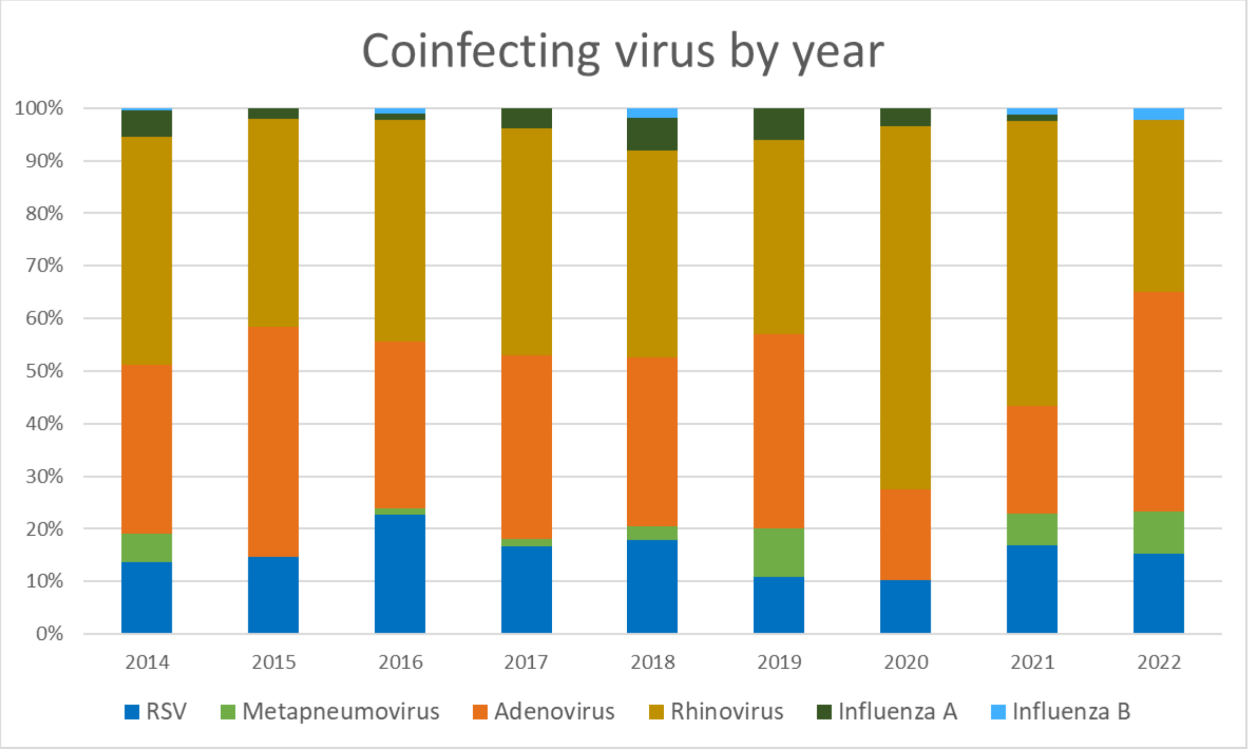

Figure S1: Co-infecting viruses in HPIV positive samples shown as a percentage of the total, by year: rhinovirus, adenovirus and RSV were consistently the most common co-pathogens in all years.

Figure S2: Seasonal epidemiology of HPIV compared with other respiratory viruses 2020-2022: (absolute numbers of positive samples by virus)

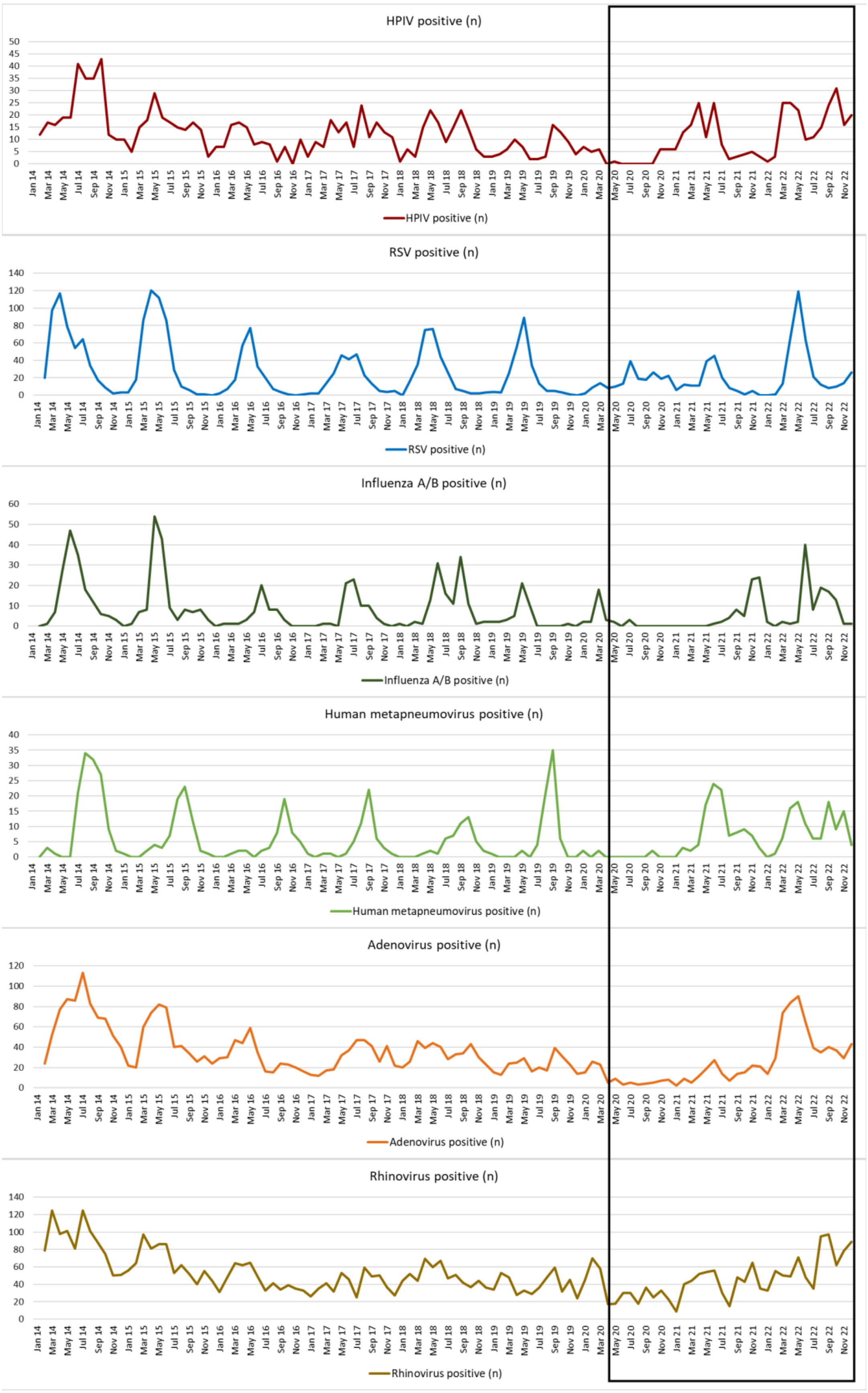

Table S1

Sample positivity rates for respiratory viruses by year:

Year	Year total samples	Year total pos	Year total pos%
2014	2567	1840	71,7%
2015	2227	1490	66,9%
2016	1634	962	58,9%
2017	1531	972	63,5%
2018	1907	1156	60,6%
2019	1618	896	55,4%
2020	1444	633	43,8%
2021	1775	828	46,6%
2022	2475	1552	62,7%

Note: Additional viruses not detected in the Allplex Essential assay are subtracted from the samples tested before March 2020 to make data comparable.

Table S2

Median patient age at testing by virus detected by year:

	Para	RSV	Flu A	Flu B	Meta	Adeno	all samples
2016	1,15	0,45	0,79	1,67	0,80	0,98	0,73
2017	0,95	0,36	0,93	0,96	0,84	1,02	0,76
2018	0,85	0,41	1,43	2,82	0,63	1,18	0,69
2019	0,68	0,28	1,61	n/a	0,61	1,17	0,61
2020	0,78	0,35	1,05	4,47	0,96	1,31	0,61
2021	1,00	0,44	1,01	1,76	0,59	1,29	0,80
2022	0,76	0,60	1,91	2,76	0,87	1,25	0,84
2016-2019	0,90	0,36	1,22	2,04	0,67	1,08	0,70
IQR	0,45-1,95	0,14-0,96	0,61-3,74	0,61-4,35	0,39-1,44	0,57-2,05	0,23-2,15
2020-2022	0,82	0,48	1,38	2,09	0,79	1,28	0,78
IQR	0,41-1,53	0,17-1,40	0,79-2,99	0,68-4,02	0,28-1,98	0,73-2,29	0,22-2,46

Table S3

Primers used for HN sequencing (globular head region):

Region	PCR reaction	Primer name	Sequence	Size (bp)	Position as per NC_021928	Reference
HN gene	Outer	HPIV4(F1)	CCCAATTTTATTCCAACTGC	789	8002-8021	This study
HN gene	Inner	HPIV4(F2)	CCAACTGCTACAACTCC	777	8014-8030	This study
HN gene	Hemi-nested	HPIV4(R)	GGGCCACCAGCTGGATCC		8790-8773	This study
